# An Intrabody Drug (rAAV6-INT41) Reduces the Binding of N-Terminal Huntingtin Fragment(s) to DNA to Basal Levels in PC12 Cells and Delays Cognitive Loss in the R6/2 Animal Model

**DOI:** 10.1155/2016/7120753

**Published:** 2016-08-10

**Authors:** I. Alexandra Amaro, Lee A. Henderson

**Affiliations:** Vybion Inc., P.O. Box 4030, Ithaca, NY 14852, USA

## Abstract

Huntington's disease (HD) is a fatal progressive disease linked to expansion of glutamine repeats in the huntingtin protein and characterized by the progressive loss of cognitive and motor function. We show that expression of a mutant human huntingtin exon-1-GFP fusion construct results in nonspecific gene dysregulation that is significantly reduced by 50% due to coexpression of INT41, an intrabody specific for the proline-rich region of the huntingtin protein. Using stable PC12 cell lines expressing either inducible human mutant huntingtin (mHtt, Q73) or normal huntingtin (nHtt, Q23), we investigated the effect of rAAV6-INT41, an adeno-associated virus vector with the INT41 coding sequence, on the subcellular distribution of Htt. Compartmental fractionation 8 days after induction of Htt showed a 6-fold increased association of a dominate N-terminal mHtt fragment with DNA compared to N-terminal nHtt. Transduction with rAAV6-INT41 reduced DNA binding of N-terminal mHtt 6.5-fold in the nucleus and reduced nuclear translocation of the detected fragments. Subsequently, when rAAV6-INT41 is delivered to the striatum in the R6/2 mouse model, treated female mice exhibited executive function statistically indistinguishable from wild type, accompanied by reductions in Htt aggregates in the striatum, suggesting that rAAV6-INT41 is promising as a gene therapy for Huntington's disease.

## 1. Introduction

Huntington's disease is an autosomal dominant inherited disease that results in the progressive loss of both motor and cognitive function for which there is no disease modifying therapy. Disease is directly linked to the expansion of a region in the huntingtin gene rich in CAG nucleotides, encoding the amino acid glutamine, that potentially results in both loss and gain of function. Mutant huntingtin protein (mHtt) degradation produces N-terminal fragments containing the polyglutamine (encoded by repeated CAG triplets) expansion that are found compartmentalized throughout the cell, particularly in the nucleus where it is linked to pathogenesis via gene dysregulation [1–5]. Data from several animal models confirm that N-terminal fragments are toxic, but the results differ with respect to which N-terminal fragments are responsible [1, 3, 5–8]. Reports demonstrating direct binding of mHtt to DNA and cellular proteins, such as transcriptional regulatory factors, suggest a direct mechanism for gene dysregulation by toxic nuclear fragments [2, 4, 9]. However, understanding the relationship between Htt fragments and human disease is often confounded by the variability observed in the ability of animal models to recapitulate human disease [10, 11].

Intrabodies, antibody fragments with intracellular function, have been described by many laboratories as a potential drug class for treating diseases, including Huntington's disease as recently reviewed by Ali et al. [12]. Several reports in both cell-based and animal models demonstrated the efficacy of one of these intrabodies, Happ1, for the potential treatment of Huntington's disease [13, 14]. Our evaluation of Happ1 in both* in vitro* and* in vivo* systems revealed low solubility (<10%) in the cytoplasm. This led us to develop a panel of alternative intrabodies with the same specificity using an intrabody-specific platform technology. Intrabodies were selected to exhibit superior cytoplasmic solubility and target engagement under the reducing conditions of the cytoplasm [15–17].

One of several selected single-chain intrabodies, INT41, that targets the proline-rich region (PRR) on the carboxyl (3′) side of the polyglutamine expansion was employed to test the role of N-terminal huntingtin protein fragments in disease. The PRR is a site of protein-protein interactions which may play a role in the linkage of N-terminal fragments to disease pathology and has been shown to affect the conformation of the huntingtin protein, particularly within the polyglutamine expansion [18, 19]. INT41 binds to a recurring proline-X-proline epitope (identical to Happ1). We show that INT41 interferes with intracellular biochemical events linked to pathogenesis and progression of Huntington's disease in cell culture and the R6/2 mouse model.

## 2. Methods

HEK293T flow cytometry: HEK293T cells (ATCC, Manassas, VA) plated at 1.2 × 10^5^ cells per well in 24-well plates were grown to 60% confluency and transfected with 0.2 *μ*g of either mHDexon-1-GFP (Q103) or HDexon1-GFP (Q30) (vectors kindly provided by Patterson as described in [13, 14]) alone or in combination with 0.8 *μ*g of either pOptivec-INT41 or pOptiVec-Happ1 plasmid using Lipofectamine (ThermoFisher Scientific) per manufacturer's instructions. After 40 hours, cells were prepared for flow cytometry analysis on a BD FACS Aria. 60,000 cells for each treatment were analyzed using the GFP filter. Resulting GFP intensities were gated into three categories (<5, 5–100, and >100).

HEK293T RNA expression: HEK293T cells were plated in 6-well plates at 0.3 × 10^6^ cells per well and grown overnight to approximately 70% confluency before transfection using Lipofectamine (ThermoFisher Scientific) with 0.8 *μ*g mHDexon-1-GFP alone or with 3.2 *μ*g of pOptiVec-INT41 plasmid. pOptiVec-INT41 alone was transfected as an additional control. Cells from quadruplicate wells were harvested with Trypsin-EDTA, centrifuged and mRNA-isolated using an Oligo-mRNA isolation kit as described by the manufacturer (Qiagen), sent to OneArray/Phalanx Biotech Group (San Diego, CA) for QC analysis, and utilized in a hybridization-based array against 29,000 genes in triplicate. Results were analyzed using Rosetta Biosoftware and statistical significance was performed using a *t*-test. Pathway analysis was also performed by Ingenuity (Qiagen).

Recombinant AAV production: rAAV6-GFP and rAAV6-INT41 were produced by Virovek (Hayward, CA) using a baculovirus system [20]. GFP or INT41 cDNA gene sequences were placed downstream from a cytomegalovirus (CMV) promoter and a human growth hormone (hGH) intron. The SV40 polyadenylation sequence (SV40 polyA) is downstream of the INT41 cDNA. The entire expression cassette is flanked by AAV6 inverted terminal repeat sequences (ITRs) required for vector packaging. After coinfection and amplification, the cell pellet was collected, lysed, cleared, and subjected to 2 rounds of cesium chloride ultracentrifugation. The rAAV vectors were buffer-exchanged to PBS buffer containing 0.001% Pluronic® F-68. rAAV vector preparations contained 2 × 10^13^ vp/mL as determined by quantitative real-time PCR.

PC12 studies and subcellular fractionation: Stable PC12 cell lines with inducible full-length human huntingtin genes with 23 CAG repeats (Q23, nHtt) or 73 CAG repeats (Q73, mHtt) were obtained from the Coriell Cell Repository and maintained on collagen-coated plates. MOI for rAAV transduction was determined by titrating rAAV6-GFP on PC12 cells to achieve approximately 99% transduction efficiency. PC12 cells were transduced with rAAV6-INT41 at an MOI of 5 × 10^5^ or carrier buffer control for 72 hours and then induced with 10–20 mM Ponasterone A (Enzo/Axxora ALX 370-014) or buffer control. At various time points, cells were harvested with Trypsin-EDTA, then washed and resuspended in lysis buffer (50 mM Tris pH 7.4, 1 mM DTT, protease inhibitors (Sigma P8340, 1 : 100) 120 mM NaCl, 0.5% NP40, and 1 mM EDTA), and cleared by microcentrifugation, and protein concentration was determined by BCA (ThermoFisher Scientific). For subcellular fractionation, buffers from a subcellular fractionation kit for cultured cells were used and samples fractionated as described by manufacturer (ThermoFisher Scientific 78840) at 8 days after induction. Protein concentration for all subcellular samples was determined by BCA (ThermoFisher Scientific).

12 *μ*g of protein from lysate or 10 *μ*g of protein from subcellular fractions was loaded and run on a NuPAGE 3–8% Tris-Acetate gel (Life Technologies EA0375) at 30 mAmps in cold 1x NuPAGE Tris-Acetate running buffer (Life Technologies LA0041) and transferred to nitrocellulose membrane in Towbin buffer at 30 V for 2 hours. Membranes were blocked in TBST/5% nonfat dry milk at room temperature (RT) for 1 hour (or overnight at 4°) before probing overnight at 4° with primary anti-N-terminal Htt polyclonal antibody (Sigma H7540), anti-actin (Genscript A00702), or a rabbit polyclonal antibody to INT41 produced by ProSci (Poway, CA) and affinity-purified at Vybion on an immobilized INT41-agarose column. All subcellular fractions were probed with antibodies to GADPH for cytoplasmic (Genscript A00084), CREB for nuclear soluble (Abcam 113622), and histone H3 for chromatin/DNA (Abcam ab1791) markers to establish the integrity of fractionation. After three washes in TBST, membranes were probed with an appropriate HRP-conjugated secondary antibody (Jackson Immunoresearch 111-035-000/115-035-174) for 1 hour at RT and then washed three times in TBST. Membranes exposed on Carestream BioMax film (VWR IB8689358) following incubation with ECL 2 chemiluminescent substrate (ThermoFisher Scientific PI80197).

R6/2 animal studies: R6/2_Tg mice and wild type (WT) littermates were bred by PsychoGenics (Tarrytown, NY) in accordance with National Institutes of Health Guidelines for the Care and Use of Laboratory Animals. PsychoGenics carried out all animal studies, ensuring the integrity of the data through a blinded quality control process. rAAV6-INT41 was dissolved in 0.1 M PBS containing 0.001% Pluronic F-68 (Sigma Cat# P5556) prior to administration. At 5 weeks of age, rAAV-INT41 or rAAV-GFP was administered bilaterally into the striatum using stereotaxic injection techniques at a dose volume of 1.5 *μ*L/injection site at a rate of 0.1 *μ*L/min. Each animal received a total of 3 *μ*L of rAAV6 virus or vehicle control (VEH). At this infusion rate, the microinjection pump delivered 1.5 *μ*L per hemisphere over 15 minutes. Due to the gender ratio of the litters obtained during breeding, each group consisted of *n* = 5 females and *n* = 9 males, with the exception of the rAAV6-INT41 group which included an additional R6/2_Tg female for a total of *n* = 6 females and *n* = 9 males.

Procedural T-maze: At 9-10 weeks, mice were tested in two T-mazes constructed of black Plexiglass (built in-house at PsychoGenics, Inc.). Each T-maze was located in a separate test room, dimly lit and equipped with a videocamera (mounted above the T-maze) and a computer and monitor. The monitor screen was covered with a red transparent film to minimize light emission. The T-maze was filled with water at 25° + /−1°, colored with Tempura nontoxic white paint to render it opaque. At one end of the cross-piece of the “T,” a platform was located approximately 0.5 cm below the level of the water. Mice were placed in the stem of the T-shaped maze and allowed to make a choice to swim into either the right or left arm to reach the escape platform. A choice was defined as entry into either the left or the right arm, without necessarily reaching the escape platform. Failure to leave the stem of the T-maze was defined as “no-choice.” Any mouse that failed to reach the platform within the maximum trial duration (60 seconds) was placed directly onto the platform. In all cases, once an animal reached or was placed on the platform, the animal was allowed to remain there for 30 seconds and was then placed back into a prewarmed holding cage allowing the fur to dry between blocks. Each mouse was trained for 8 trials per day. Testing was conducted in blocks of approximately 8 mice, such that every mouse performed one trial before returning to the first mouse for the second trial. Thus, intertrial intervals were maintained at approximately 15 minutes. Mice were tested for 6 days per week, for a maximum of 2 weeks (total of 12 sessions). Acquisition of the task was defined as 75% correct for two consecutive days. For the procedural T-maze data, acquisition data were analyzed using Statview software (SAS Institute Inc.) by a two-way ANOVA with the factors gender and genotype-treatment (4 levels). In addition, the proportion of mice successfully acquiring the task each day was analyzed via Kaplan-Meier analysis.

*Postmortem Analysis*. Following the completion of testing at 12 weeks of age, animals were anesthetized using Sodium Pentobarbital i.p. (100 mg/kg BW) and transcardially perfused using a peristaltic pump with 4% paraformaldehyde (PFA) in 0.1 M PBS (pH = 7.4) on ice. Brains were then removed from skull and postfixed overnight at 4° and stored at 4° in 0.1 M PBS containing 0.01% Sodium Azide until the time of shipment to NeuroScience Associates (NSA; Knoxville, TN) that prepared brain sections and performed the analysis of aggregates. Microscopic evaluation of mouse brains was prepared using the MultiBrain technology and stained with EM-48 for Htt aggregates (IHC). Approximately 28 coronal sections were evaluated for changes in treated groups and compared to controls. The enumeration of aggregates was determined at two levels per animal and performed identically across all animals with a 40x objective. Both striata were imaged at two different coronal levels, 4 images per animal, and analyzed as described by Simmons et al. [21] using Image-Pro Plus software (MediaCybernetics). Aggregates were binned by size per animal and spreadsheet arrayed data was analyzed by frequency analysis for the incidence of specified sizes (Figure 5). To determine the significance of differences in numbers of aggregates below 1 *μ*m, analysis of variance was used (Figure 5).

## 3. Results

**Figure 1:**
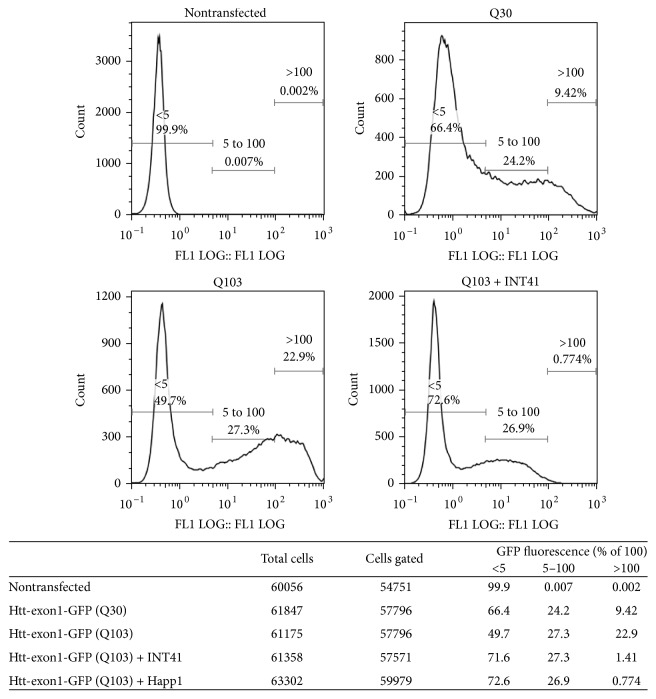
INT41 reduces Htt-exon1-GFP (Q103) aggregation in HEK293T cells. HEK293T cells were transfected with vectors encoding Htt-exon1-GFP with either a short (Q30) or extended (Q103) glutamine repeat region. Vectors encoding the intrabodies Happ1 or INT41 were cotransfected with Htt-exon1-GFP (Q103). After 40 hours, flow cytometry analysis on a FACS Aria was performed and GFP signals were gated into three categories based on GFP intensity. Htt aggregates are represented in the >100 signal category. The percentage of cells in each category for each treatment is shown.

The intrabody INT41, specific to the PRR region of the huntingtin protein (Htt), was developed and tested in parallel to Happ1 [13, 14]. During our initial characterization in HEK293T cells cotransfected with INT41 or Happ1, along with the first exon of the huntingtin gene with 103 CAG repeats (Htt-exon1-GFP (Q103)), we observed that both intrabodies reduced aggregation of transiently overexpressed mHtt by flow cytometry analysis (Figure 1). Since Htt fragments have been shown to bind to DNA and are also associated with gene dysregulation (see Section 1), we extended our initial observations with RNA expression analysis. Cells expressing INT41, Htt-exon1-GFP (Q103), or both were harvested, mRNA-extracted, and subsequently analyzed by hybridization against 29,000 genes. Hundreds of genes were dysregulated by expression of Htt-exon1-GFP (Q103). When the array data was analyzed by Integrity Pathway analysis, 1005 genes exhibited gene dysregulation by mHtt expression with a *p* value of <0.05 and 88 genes had a *p* value of <0.001. A representative subset of these results are displayed in Table 1. INT41 alone did not broadly affect gene expression, with the exception of a few genes required to express and degrade proteins. Coexpression of INT41 with Htt-exon1-GFP (Q103) suppressed altered gene expression broadly by about 50%. Although Integrity Pathway Disease analysis included Huntington's disease as an affected pathway, the breadth of gene dysregulation suggests that gene dysregulation by Htt-exon1-GFP (Q103) is nonspecific with respect to any group of genes or pathways, consistent with observations from other laboratories (reviewed by [22, 23]).

**Figure 2:**
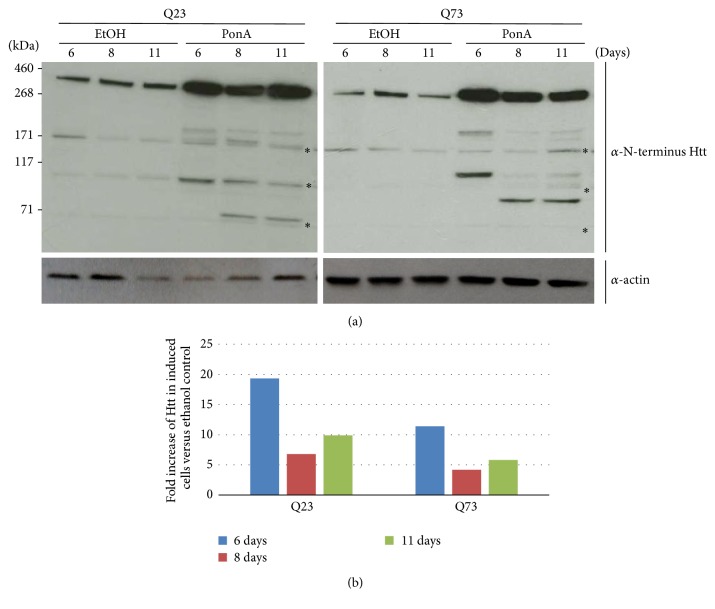
Time course demonstrating expression and degradation of induced Htt in PC12 cells: (a) PC 12 cells with inducible Htt (either nHtt (Q23) or mHtt (Q73)) were cultured in collagen-coated 12-well plates and induced with Ponasterone A (PonA) or carrier buffer (EtOH). Wells were harvested at the indicated days after induction. Precleared lysates (12 *μ*g) were run in each lane on a 3–8% Tris-Acetate gel, transferred to nitrocellulose, and immunoblotted with rabbit anti-N-terminal Htt antibody and secondary antibody as described (Section 2). Endogenous rat Htt bands are identified by the asterisk (*∗*). The same membrane was reprobed using an antibody against actin to serve as a loading control. (b) Fold increase of full-length Htt in induced cells compared to vehicle control as determined by ImageJ analysis.

Since dual transfection has variable efficiency, further experiments were carried out using rat PC12 cells engineered using the RheoSwitch system, in which full-length human Htt could be induced. Furthermore, PC12 cells can be transduced with an adeno-associated virus vector carrying the coding sequence for the intrabody INT41 (rAAV6-INT41) with high efficiency. To establish that two cell lines, PC12-Q23 (nHtt, Q23) and PC12-Q73 (mHtt, Q73), produced comparable quantities of Htt with similar kinetics, we induced both cell lines with Ponasterone A (PonA) and harvested cells at multiple time points. Precleared lysate samples were run on gels, transferred to nitrocellulose, and probed with an anti-N-terminal Htt antibody (Figure 2). Endogenous rat Htt was detected in the EtOH control samples by the N-terminal Htt specific antibody, most likely due to sequence similarity between human and rat Htt. Induced full-length 350 kD human huntingtin protein was expressed at 5–20 times the level of endogenous rat huntingtin protein (ImageJ digital analysis) consistently in multiple experiments and at multiple time points from 1 to 11 days. Shifts in smaller bands specific to induced human N-terminal Htt bands reflect the 7 kD difference in size between Q23 and Q73, which is evident as the degradation fragments become smaller (Figure 2). Endogenous rat Htt bands were identified as those that did not change in intensity between control (EtOH) and induced (PonA), as well as those bands in the lower range of the gel that did not alter migration to account for the 7 kD difference in molecular weight between Q23 and Q73.

**Figure 3:**
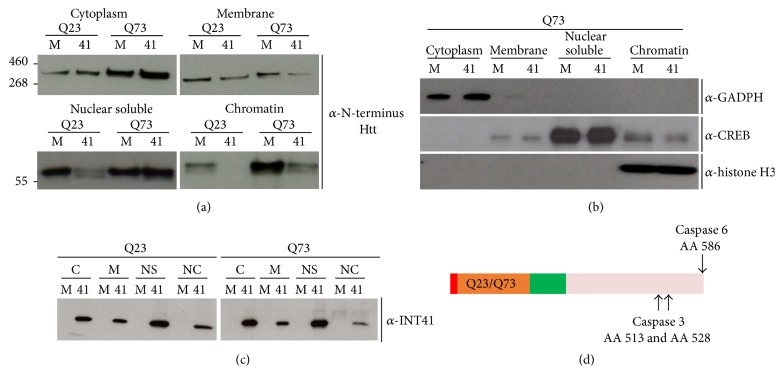
INT41 reduces binding of mHtt fragments to chromatin in PC12 cells: PC12 cells (nHtt Q23 and mHtt Q73) were transduced with rAAV6-INT41 (41) or mock-transduced (M) and then induced for Htt expression after 72 hours with Ponasterone A. Cells were harvested 8 days after induction and then subjected to subcellular fractionation as described (Section 2). 10 *μ*g protein from each fraction was separated on a 3–8% Tris-Acetate gel, then transferred to nitrocellulose, and (a) immunoblotted with rabbit anti-N-terminal Htt antibody and secondary as described (Section 2). (b) Immunoblot with a mouse anti-CREB, anti-GADPH, or anti-Histone H3 as markers for subcellular fractionation. Molecular weights are in kilodaltons as indicated. (c) INT41 distribution into subcellular compartments. A blot from subcellular distribution was reprobed with affinity purified rabbit anti-INT41 to obtain intrabody distribution between cytoplasmic (C), membrane (M), nuclear soluble (NS), and nuclear chromatin (NC) fractions for both mock- (M) and rAAV6-INT41-transduced cells (41). (d) Schematic representation of Caspase 3 and Caspase 6 cleavage of Htt.

We used subcellular fractionation to see if we could discern differences in Htt fragment distribution between mock and rAAV6-INT41-transduced cells. In seven independent experiments, we first observed a dominating doublet of approximately 64 kD recognized by an N-terminal anti-Htt antibody in the chromatin/DNA (micrococcal nuclease treated to release DNA bound protein) fraction of induced Q23 and Q73 PC12 cells (Figure 3(a)). The similar molecular size of the N-terminal Htt fragment(s) of Q73 and Q23, despite the difference in the glutamine repeat region, could arise by caspase cleavages (Figure 3(d)), which are associated with increased expression of Htt with an extended polyQ region ([23, 24], see Section 4). As a result of differential caspase cleavage, the carboxyl termini of these fragments may differ. The N-terminal mHtt signal associated with chromatin/DNA was 6-fold higher (ImageJ digital analysis) than the corresponding N-terminal nHtt signal (Figure 3(a), Chromatin Q73 lane M versus Q23 lane M). Induced Q73 cells transduced with rAAV6-INT41 exhibited a 6.5-fold reduction of the 64 kD doublet in the chromatin/DNA fraction when digitally analyzed by ImageJ (Figure 3(a) Chromatin Q73 lane M versus lane 41). This suggests that mHtt has a higher affinity for binding DNA and that the increased binding is blocked by INT41. A similar reduction of nHtt (Q23) binding to chromatin in the presence of INT41 was also observed. A decrease in N-terminal nHtt fragments in the nuclear soluble fraction for Q23 cells expressing INT41 (Figure 3(a)) may account for the decrease of nHtt binding to chromatin. There is no corresponding increase in N-terminal mHtt fragments in the nuclear soluble fraction of cells also transduced by rAAV6-INT41 when chromatin/DNA binding is reduced (Figure 3(a) Nuclear Soluble Q73 lane M versus lane 41) suggesting that INT41 may also have a role in slowing translocation of nHtt and mHtt to the nucleus, though this would have to be further investigated. However, in several experiments the 64 kD doublet was also reduced in the nuclear soluble fractions in cells transduced with rAAV6-INT41 versus mock transduced cells. Antibodies representing proteins unique to a particular fraction were used to validate the integrity of the subcellular fractionation (Figure 3(b)). To verify that INT41 was expressed in these subcellular fractions, blots were stripped and reprobed with anti-INT4 (Figure 3(c)). As expected, INT41 was observed only in transduced cells. INT41 was observed in all fractions, with the least amount in the nuclear/chromatin fraction of PC12-Q73 cells.

**Figure 4:**
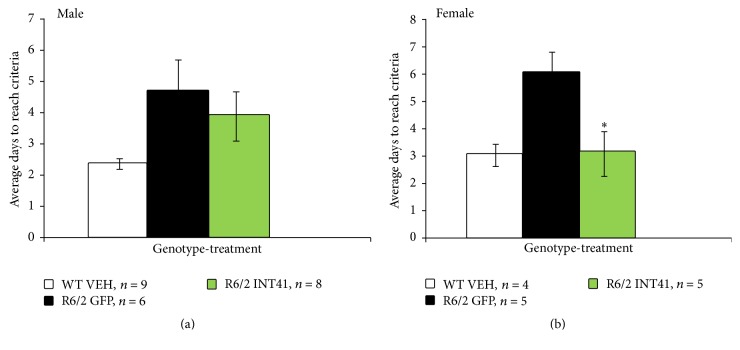
INT41 stabilized cognitive function in Huntington's disease R6/2 animal model: mice received bilateral intrastriatal injections of rAAV6-INT41 (R6/2 INT41) or rAAV6-GFP (R6/2 GFP) and wild type mice received vehicle injection (WT VEH) as described in Section 2. At 9-10 weeks, mice were subjected to the T-maze swim test daily for 6–12 days to measure memory acquisition (see Section 2). The *∗* denotes statistical significance of the exhibited cognitive function that is indistinguishable from vehicle-treated control wild type mice as determined by a two-way ANOVA.

To examine the potential efficacy of INT41 in a gene therapy strategy, striatal delivery of rAAV6-INT41 in the R6/2 model was used in conjunction with cognitive and motor function testing (performed by Psychogenics, Tarrytown, NY). Animals are typically born with demonstrable aggregates in neurons and show behavioral and cognitive dysfunction within 3-4 weeks during a life span of 12–14 weeks. Mice received bilateral intrastriatal injections at 5 weeks of age and testing was performed at 9-10 weeks of age. rAAV6-GFP was used as a control and IHC staining of brain sections for GFP served as a surrogate to determine the efficiency of both intrastriatal delivery and transduction. Using the T-maze test as an established test for cognitive function, we recorded when mice could demonstrate on consecutive days the ability to reach an escape platform (see Section 2). We observed that female mice, which typically exhibit disease progression two weeks slower than males as determined by their decline in cognitive and motor function, treated with rAAV6-INT41 exhibited cognitive function indistinguishable from vehicle-treated control wild type mice (Figure 4). Male mice treated with rAAV6-INT41 also trended toward better cognition as compared to controls, but the differences were not statistically significant.

**Figure 5:**
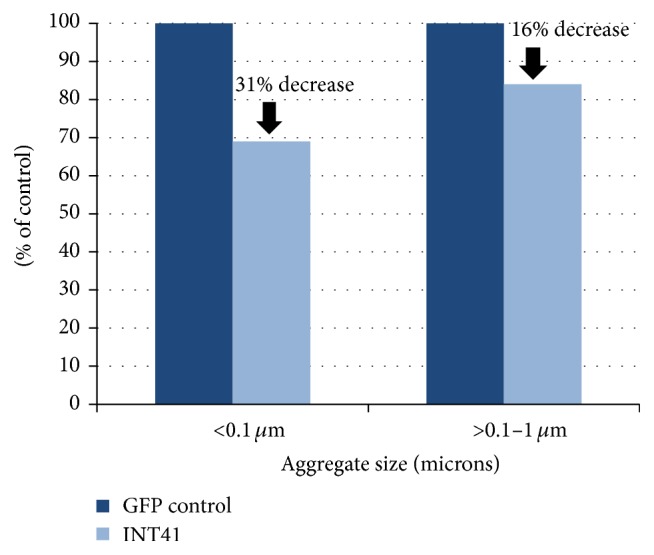
INT41 reduced Htt aggregate formation in R6/2 animal model: aggregates in coronal sections of female mice were measured in 4 fields, binned by size, and analyzed for the frequency of sized aggregates as indicated. Analysis of variance on 4113 data points for 0-1 *μ*m size category was 0.085 comparing striatal fields, as described in Section 2, from 5 GFP and 6 INT41 mice.

We subsequently used the scanning methods and data acquisition as described by Simmons et al. [21] to evaluate aggregate formation in the striatum of IHC-stained brain sections with the EM48 antibody. Distribution of rAAV6, as determined by anti-GFP-HRP histology, was found predominately in the putamen and caudate as well as the thalamic regions of the brain. Frequency analysis was used to evaluate the incidence of various sizes of striatal Htt aggregates within the INT41 and control (GFP)-treated animals. There was a 31% decrease in the frequency of smaller aggregates (<0.1 mm) in the INT41 (*n* = 6) versus GFP (*n* = 5) female treatment groups and a 16% decrease in the formation of aggregates between 0.1 and 1 *μ*m (Figure 5). Taken together, decreases in the incidence of aggregates in mice as a result of expression of INT41 could also improve cognitive function.

## 4. Discussion

We have utilized both cell culture and an* in vivo* mouse model to better understand the mHtt protein in disease progression as well as the effect of the intrabody INT41 on that protein. The data in Figure 2 confirms N-terminal degradation fragments reported previously by numerous investigators using PC12 or other cell lines [1, 3, 5, 6]. Cell lysis conditions using the nonionic detergent NP-40 in low salt conditions leave the nuclei intact, which are separated by centrifugation from the soluble lysate by pelleting with the insoluble cellular debris. Hence, the smaller N-terminal fragments found in the nuclear fraction in Figure 3 are in the insoluble fraction and do not appear on the blot consisting of the soluble fraction only. However, Figure 2 confirms the degradation of Htt into multiple N-terminal fragments that are associated with cellular toxicity.

Several studies have demonstrated an association of N-terminal Htt fragment nuclear toxicity and aggregation to the interaction with DNA, often aggregating with transcription factors [1–4]. Our results confirm and extend these findings by demonstrating an interaction dominated by a specific Htt fragment with DNA as a function of polyQ length (Figure 3) and provide a biochemical link to gene dysregulation and pathogenesis. Although prior reports localized N-terminal fragments to a nuclear location [1, 3, 4], this is the first report using subcellular fractionation validated with biomarkers for subcellular compartments that localize specific Htt fragments to the nuclear fraction. The results also provide a mechanism for the ability of INT41 to suppress gene dysregulation by inhibiting binding of N-terminal fragments to DNA which is likely charge dependent.

There are multiple intracellular events linked to the mutant huntingtin protein, including gene dysregulation, which may be a common feature of similar neurodegenerative diseases as well (recently reviewed by [22]). Our data is consistent with Htt fragment DNA binding and possible aggregation in the nucleus due, in large part, to the ionic interaction of the positively charged and solvent exposed polyQ region with negatively charged DNA that increases in avidity with polyQ length. As such, it is likely that gene dysregulation is nonspecific, involving multiple interactions with elements of DNA (enhancers, activators, repressors, promoters, noncoding regulatory sequences, etc.) as well as transcription factors. The results in Figure 2 also suggest that there may be a preferred conformation for DNA binding, reflected in the increased presence of mHtt (Q73) in the chromatin fraction. There may be differences between nHtt and mHtt in accessibility to proteases, since the fragments of N-terminal mHtt and nHtt are of roughly equivalent molecular size. If these were the same proteolytic fragment, their size should differ by approximately 7.25 kD (50 glutamines). Prior work from multiple laboratories has shown that Caspase 3 is produced in response to mHtt as part of apoptosis and/or pathogenesis [23, 24]. Caspase 3 cleaves Htt at residues 513 and 528 (Figure 3(d)). Proteolysis of a 586-amino acid protein fragment (resulting from Caspase 6 cleavage) would reduce mHtt by 7-8 kD accounting for this similar migration. Since Caspase 3 cleavage efficiency is also dependent on polyQ length [23], conformational differences between nHtt and mHtt produced by polyQ length are likely since the amino acid sequence between nHtt and mHtt is otherwise identical with the exception of polyQ sequences that are over 400 amino acids from the Caspase 3 site.

It is notable that we did not detect binding to DNA by other N-terminal fragments (Figures 2 and 3), although continued proteolytic degradation would generate multiple fragments of shorter lengths [13]. However, affinity chromatography with cell lysates run over an immobilized INT41-sepharose column did reveal INT41 binding to smaller N-terminal fragments, but their localization was not determined (data not shown). Affinity purification using INT41 also did not demonstrate binding to full-length Htt, suggesting that the epitopes are either cryptic or bound to accessory proteins. It is also notable that INT41 inhibition of mHtt fragment binding to DNA did not increase levels of the 64 Kd doublet in the nuclear soluble fraction (Figure 3), suggesting that INT41 may reduce trafficking of these N-terminal fragments to the nucleus, in addition to reducing the binding of N-terminal fragments that do gain entry into the nucleus. Our results (Figure 3(a)) show reduction of both Q23 and Q73 Htt fragments in the nuclear soluble fraction.

Several lines of investigation have shown an association between protein aggregation and secondary structure of the N-terminal region of mHtt as a function of polyQ length as a driver of the aggregation process [18, 25–29], but questions still remain as to the nature and function of the toxic aggregate, soluble aggregate or complex, and its mechanism of pathogenesis [30–32]. The most compelling association is the fact that increasingly longer polyQ length is directly associated with earlier age of disease onset and a faster rate of disease progression in humans. Binding of N-terminal fragment(s) of mutant huntingtin protein to DNA is a likely cause of widespread gene dysregulation and may be a significant factor in disease progression and neuron loss by induction of apoptosis.

Gene dysregulation precedes symptoms of human Huntington's disease by up to 15 years, affecting hundreds of genes [33–36]. Disruption of gene regulatory networks can have profound effects on cellular function [37, 38]. Benn et al. [2] used R6/2 striatum, human HD postmortem patient samples, and Htt-expressing cell lines to examine polyQ-dependent Htt binding directly to DNA and transcription factors using gene expression, ChIP, and transcription factor arrays. Our results extend those findings by identifying a specific dominating N-terminal fragment interaction with DNA that may drive broad dysfunction in neurons, although it remains possible that there are other macromolecular interactions involved.

AAV6 was used for gene delivery based on reports of AAV6 retrograde transport favoring striatal delivery and neuron specificity for Huntington's disease [39]. R6/2 is a transgenic mouse model with 120 CAG repeats and is the best characterized of the R6/1 and R6/2 Htt-exon1 transgenics [40]. The R6/2 data showing inhibition of the formation of smaller aggregates (Figure 5) is consistent with INT41 stabilizing soluble mHtt fragments as well as reducing the formation of early microaggregates (Figure 5). Further, the R6/2 data (Figures 4 and 5) demonstrate a direct correlation between INT41, reduction of microaggregate formation in the striatum, and the delay in loss of executive function. Ninety-five percent of human patients have polyQ repeats of less than 70. Our results in PC12 with mHtt (Q73) and R6/2 with mHtt (Q120) suggest potential efficacy within this range of polyQ expansion. However, as polyQ increases in length, the charge-charge interaction between N-terminal mHtt fragments and DNA will increase.

In summary, our data suggest that INT41 alters the direct consequences of the N-terminal mHtt interaction with chromatin/DNA and support a link of these N-terminal Htt fragments to gene dysregulation. INT41 reduces the binding of N-terminal mHtt fragments to nuclear chromatin/DNA to levels observed in mock-transduced nHtt cells and lowered the compartmental concentration of N-terminal Htt fragments in the nuclear soluble fractions. In the R6/2 animal model, INT41 alleviates loss of cognitive function and reduces aggregate formation in the striatum. The ability of INT41 to interfere with gene dysregulation by Htt, a mechanism that drives pathogenesis of Huntington's disease, makes this an attractive therapeutic strategy. Gene dysregulation is a common feature of polyglutamine expansion diseases and may be a major driver of progression and pathogenesis [22, 41, 42]. INT41 may be therapeutic in similar PolyQ diseases where there is an adjacent proline-rich region containing target sequences (SMA, SCA 1, SCA 3, and SCA 7).

## Figures and Tables

**Table 1:** Gene dysregulation by Htt-exon1-GFP (Q103) reduced by INT41: (1 & 3) fold change in gene expression versus control HEK293T adjusted for weighted error (NC = no change). (2) fold change in expression versus Htt-exon1-GFP (Q103) adjusted for weighted error (numbers are relative to nontransfected HEK293T with a value of 1).

Gene	Q103^1^	Q103 + INT41^2^	INT41^3^	Comments
NRCAM	0.44	1.80	NS	Neuronal CAM
IMMT	0.32	1.66	NS	Mitochondrial IM protein
ANXA4	0.36	1.58	NS	Endo/exocytic
PCDH9	0.37	1.68	NS	Neuronal protocadherin
BNIP3	0.38	1.91	NS	Proapoptosis
NRP1	0.45	1.92	NS	Neurophilin
UBE2E3	0.59	1.30	NS	Ubiquitin conjugating enzyme
UBE2J2	6.32	0.58	2.7	Ubiquitin conjugating enzyme
HSPA1B	5.88	0.56	NS	HSP701B stabilizes aggregation
PDF	8.66	0.49	1.97	Protein synthesis regulator
SPCS1	4.20	0.55	NS	Ubiquitin signal peptidase
NCSTN	4.17	0.57	NS	Notch/beta amyloid cleavage (AZ)
CTSD	4.07	0.37	NS	Aspartyl protease (AZ)
HSP90AA	2.79	NS	NS	Cytosolic HSP90 alpha
